# Genomic correlates of extraintestinal infection are linked with changes in cell morphology in *Campylobacter jejuni*

**DOI:** 10.1099/mgen.0.000251

**Published:** 2019-02-19

**Authors:** Nicole E. Wheeler, Timothy Blackmore, Angela D. Reynolds, Anne C. Midwinter, Jonathan Marshall, Nigel P. French, Matthew S. Savoian, Paul P. Gardner, Patrick J. Biggs

**Affiliations:** ^1^​ Center for Genomic Pathogen Surveillance, Wellcome Sanger Institute, Hinxton, UK; ^2^​ School of Biological Sciences, University of Canterbury, Christchurch, New Zealand; ^3^​ Biomolecular Interaction Centre, University of Canterbury, Christchurch, New Zealand; ^4^​ Capital and Coast District Health Board, Wellington, New Zealand; ^5^​ EpiLab, School of Veterinary Science, Massey University, Palmerston North, New Zealand; ^6^​ New Zealand Food Safety Science and Research Centre, Palmerston North, New Zealand; ^7^​ Institute of Fundamental Sciences, Massey University, Palmerston North, New Zealand; ^8^​ Department of Biochemistry, University of Otago, Dunedin, New Zealand.; ^9^​ New Zealand Genomics Ltd (NZGL – as Massey Genome Service) Massey University, Palmerston North, New Zealand; ^10^​ Allan Wilson Centre for Molecular Ecology and Evolution, Massey University, Palmerston North, New Zealand

**Keywords:** *Campylobacter jejuni*, random forest, bacteremia

## Abstract

*Campylobacter jejuni* is the most common cause of bacterial diarrheal disease in the world. Clinical outcomes of infection can range from asymptomatic infection to life-threatening extraintestinal infections. This variability in outcomes for infected patients has raised questions as to whether genetic differences between *C. jejuni* isolates contribute to their likelihood of causing severe disease. In this study, we compare the genomes of ten *C. jejuni* isolates that were implicated in extraintestinal infections with reference gastrointestinal isolates, in order to identify unusual patterns of sequence variation associated with infection outcome. We identified a collection of genes that display a higher burden of uncommon mutations in invasive isolates compared with gastrointestinal close relatives, including some that have been previously linked to virulence and invasiveness in *C. jejuni*. Among the top genes identified were *mreB* and *pgp1*, which are both involved in determining cell shape. Electron microscopy confirmed morphological differences in isolates carrying unusual sequence variants of these genes, indicating a possible relationship between extraintestinal infection and changes in cell morphology.

## Data Summary

1. Sequence data has been deposited in the Sequence Read Archive; accession number PRJNA475221 (URL - https://www.ncbi.nlm.nih.gov/bioproject/PRJNA475221).
2. Lists of orthologous genes, corresponding bitscore data and code used for this analysis have been deposited in GitHub (URL - https://github.com/Gardner-BinfLab/invasive_campylobacter).

**Impact Statement**

*Campylobacter* is the largest contributor to bacterial diarrhea in the world. Most infections are self-limiting, however, a small subset result in extraintestinal infection, which is associated with an increased mortality rate. While risk factors influencing host susceptibility have been identified, it is still unclear whether genomic factors associated with the bacteria also contribute to infection outcome. In this study, we have identified genes that have accumulated unusual mutations in isolates captured from bacteremic patients. Among them were two genes involved in determining cell shape, highlighting a possible link between cell morphology and invasive potential. Our analysis showcases a high-throughput approach to the detection of functionally important sequence variation in genes which lead to measurable differences in phenotype. However, whether these mutations contributed to, or resulted from this shift in niche remains unclear.

## Introduction

*Campylobacter jejuni* is the most common bacterial cause of diarrhea in the world [1]. Variability in the clinical presentation of infection with members of the genus *Campylobacter* has been a subject of interest for some time now [2], as clinical presentation can range from asymptomatic infection [3], to diarrhea [4], to invasive infections, such as meningitis and bacteremia [4–6]. *Campylobacter* bacteremia appears to originate from acute colitis, indicating a progression from diarrheal disease to extraintestinal infection in a subset of cases [6]. While extraintestinal infections with members of the genus *Campylobacter* disproportionately affect the very young, the very old and the immunocompromised, the wide range of disease presentations cannot be explained purely by host factors, indicating that differences in the bacterial pathogen may also contribute [2, 6, 7]. Over decades of study, cytotoxin production, motility and the ability to adhere to epithelial cells have been implicated as genetic factors in members of the genus *Campylobacter* that are associated with disease presentation [8–10]. In addition, early studies identified associations between specific genetic loci and virulence [2, 11–14], however, follow-up studies that attempted to replicate these findings have returned largely negative results [15–17]. As a result, there is still uncertainty as to which genetic factors make some isolates of members of the genus *Campylobacter* intrinsically more likely to cause more severe disease.

Members of the genus *Campylobacter* are a major cause of bacterial infections in New Zealand. New Zealand has relatively high rates of campylobacteriosis notifications compared with other countries [18], despite a major decline in human cases of campylobacteriosis in 2007/8 associated with interventions in the poultry industry [19]. In 2016 the rate of campylobacteriosis in New Zealand was 158.9 per 100, 000 people, the highest number of cases for any notifiable disease [20]. As a comparison, in the UK the rate of campylobacteriosis in 2016 was 90.8 per 100,000 [21]. Although *Campylobacter* bacteremia rates are believed to be under-detected and under-reported [10], of a total 59, 801 cases of *Campylobacter* infection in New Zealand from 2010 to the present, 167 cases (0.28 %) involved *Campylobacter* isolated from blood (P. Cressey, Institute for Environmental Science and Research Ltd, personal communication, 23 October, 2018). While precise recent rates of *Campylobacter* bloodstream infections are not available for the UK, 1,665 out of 994,791 (0.17%) of *Campylobacter* patients in England and Wales from 1989 to 2011 had infections involving bloodstream infection [22], and these rates have not changed significantly since then (G. Godbole, Public Health England, personal communication, 23 October 2018). These figures indicate a significant difference in both the rates of campylobacteriosis (*P*=2.0×10^−5^, odds ratio=1.7) and the incidence of subsequent bloodstream infection (*P*=3.6×10^−9^, odds ratio=1.7) between the two countries. This makes species of the genus *Campylobacter* infectious agents of strong relevance to public health, and indicates that there may be lifestyle and/or genetic factors of both host and pathogen that put New Zealanders at greater risk. In order to assess whether there may be genetic factors associated with extraintestinal infection in New Zealand isolates of *Campylobacter jejuni*, ten isolates of *C. jejuni* were collected at Wellington Hospital from the blood (*n*=9) or joint aspirate (*n*=1) of patients showing symptoms of *Campylobacter* bacteremia. These were compared with closely related reference strains from the Oxfordshire surveillance project (OXC), a genomic surveillance program investigating seasonal and temporal trends in members of the genus *Campylobacter* that cause gastroenteritis in Oxfordshire, United Kingdom [23].

We performed a comparative analysis to determine whether there were any genomic changes that occurred preferentially and independently in these extraintestinal isolates from different clonal complexes. We found no significant differences in gene content between extraintestinal and gastrointestinal strains, indicating that any genomic differences relating to potential to cause extraintestinal infection would either be unique to individual strains or reflected in different allelic variants of genes shared by these isolates. We next searched the genome for genes which carry an excess of rare mutations in extraintestinal isolates. Using this screening strategy, we have identified in extraintestinal isolates from our study a small number of candidate genes which accumulated mutations that are rarely observed in nature, some of which have been associated with virulence and invasiveness in the past. Our results indicate that there may be a shift in the selective pressures on genes involved in virulence, lipopolysaccharide biosynthesis, stress tolerance, iron acquisition and cell shape when *C. jejuni* transitions from gastrointestinal to invasive infection.

## Methods

In this investigation we compared ten invasive clinical isolates of *Campylobacter jejuni* with closely related gastrointestinal strains from the Oxfordshire surveillance project in order to identify genetic differences associated with extraintestinal infection. We employed a profile hidden Markov model (HMM)-based approach [24] to weight sequence variation in genes based on its predicted functional significance. We then used a random forest model to identify genes whose burden of rare mutations best separated out extraintestinal and gastrointestinal strains.

### Isolate collection

Isolates were collected from hospitals in the Wellington region from 2010 to 2012. The age of patients the isolates were collected from ranged from 19 to 89 years. Six presented with diarrhea, others presented with headache, prosthetic hip infection and exacerbation of chronic pulmonary disease. Nine isolates were taken from blood and one from joint aspirate. Six out of ten patients were subsequently treated with ciprofloxacin, one patient died, and one had had a previous invasive infection four years earlier. Only one person in the study showed sepsis syndrome. As comparators for each isolate, sequence data for two randomly selected isolates from the same clonal complex were retrieved from the Oxfordshire surveillance project collection [23] using BIGSdb [25], based on a recommended sampling strategy for identifying phenotypically relevant sequence variation in structured populations [26]. Two additional New Zealand gastrointestinal isolates were included to supplement a clonal complex not found in the Oxfordshire surveillance project (Table S1 , available in the online version of this article). For electron microscopy, contemporaneous human gastrointestinal isolates of the same sequence type (ST) isolated as part of the Manawatu sentinel surveillance project [27] were used for comparison of morphological features.

### DNA extraction and sequencing

Isolates were grown on Columbia horse blood agar plates (Fort Richard Laboratories) in a microaerobic atmosphere (85 % N_2_, 10 % CO_2_, 5 % O_2_) at 42 °C for 24 h in a VA500 variable atmosphere incubator (Don Whitley Scientific). DNA was extracted using a QIAamp DNA Mini kit (Qiagen). The resultant DNA was then sent to the Massey Genome Service (Massey University, Palmerston North, New Zealand) for quality control checking, library preparation and sequencing. The isolates were sequenced on an Illumina MiSeq (250 bp paired end run) to approximately 140×–200× estimated coverage based on the size of the *Campylobacter jejuni* genome.

### Read curation and assembly

Sequence reads were analysed with the following quality controls: read quality analysis and visualization (SolexaQA ++and FastQC) [28, 29], PhiX removal with Bowtie2 [30] and adapter removal through the ‘fastq-mcf’ program from the ea-utils suite of tools [31]. In addition, the reads were analysed with FastQScreen (http://www.bioinformatics.babraham.ac.uk/projects/fastq_screen) as a further check for any potential Illumina adapters and cloning vector contamination. For each sample, sequence reads were used to create multiple assemblies using VelvetOptimiser v2.2.5 [32] and Velvet v1.2 [33]. Assemblies were performed for kmer sizes varying between 60 and 90 % of the read length. An assembly improvement step was applied to the assembly with the best N_50_, in which contigs were scaffolded using SSPACE [34] and sequence gaps filled using GapFiller [35]. Genome Sequence data for the New Zealand isolates was deposited in the Sequence Read Archive (BioProject PRJNA475221) [Data Citation 1]. Accession numbers for all strains are listed in Table S1.

### Core genome generation

Contigs were annotated using Prokka v1.5 [36] with default parameters and a genus-specific database from RefSeq [37]. Protein-coding regions were extracted and translated, and gene clustering was performed using Roary [38]. A core gene alignment produced by Roary was then visualized in SplitsTree [39]. A full list of orthologous genes can be found in the Github repository accompanying this paper [Data Citation 2]. SplitsTree visualization indicated that the representatives that were chosen from ST50 were too distantly related to the New Zealand strains, so all samples from ST50 from the Oxfordshire Surveillance project with publicly available sequencing data were collected and assembled using Velvet. Relatedness of strains was assessed using mash [40]. Four better representatives were chosen for this ST and replaced the previously selected strains and gene clustering was repeated.

### Identification of functionally significant variation in orthologous proteins

To screen for differences in the burden of rare mutations on a gene across infection outcomes, genes were scored against HMM profile models for Epsilonproteobacterial protein sequences retrieved from the EggNOG database (v. 4.5.1) [41], using the hmmsearch function from the HMMER3.0 package (http://hmmer.org). In order to resolve many-to-many matches between EggNOG profiles and protein sequences in our dataset the EggNOG profile with the best score summed over all genes in an orthologous group was chosen as the gene model for that group. Bitscore data for all orthologous groups can be found in the file ‘bitscores’ [Data Citation 2]. Start codons for some genes were annotated inconsistently. This difference in length led to differences in bitscore that affected the analysis and led to spurious associations, so we aligned the orthologous gene families and trimmed off any gap columns at the beginning of the alignment before scoring with HMMER, and if the length trimmed exceeded 20 amino acids, the gene was discarded from this part of the analysis. We also excluded any genes that had paralogs identified by Roary, because we noted that some of these families showed arbitrary splitting of gene families into two, which also produced spurious results. Finally, we removed genes that showed little to no variation in bitscore using the nearZeroVar function in the ‘randomForest’ R package [42]. This approach resulted in 1,161 orthologous genes families with EggNOG model hits for analysis. DeltaBS, a weighting of the functional effects of mutations on protein coding genes [24], was calculated by subtracting individual bitscores from the median bitscore for an orthologous group.

### Identification of informative genes using a random forest

A random forest algorithm [43] was used to build classification trees from bitscore data for orthologous groups, using infection outcome (extraintestinal or gastrointestinal) as classes for prediction. Random forests can capture interactions between genes, have been found to be a particularly effective way of dealing with sparse data [44], and their utility in linking genotype to phenotype is being explored for these reasons [45–47]. They consist of an ensemble of recursive partitioning trees, each built using a random sub-sampling of samples and variables (genes) [43, 48]. Although random forests are primarily designed for the classification of new samples, they produce variable importance measures which indicate how informative each variable has been in separating cases according to category. These variable importance measures can be used to assess which genes may be important in identifying extraintestinal *C. jejuni* isolates.

Because the dataset was likely to be sparse, meaning that most of the genes in the training dataset offer little to no predictive value in determining the phenotype of interest, we selected a subset of genes before training the random forest model. To further enrich for potentially informative genes, we selected only genes that showed differences in median bitscore between the two groups that are in the highest 80 % [|median(BS_GI_)-median(BS_EI_)| ≥0.15], and separation of bitscore distributions, as measured by a Kolmogorov–Smirnov (KS) test that fell in the highest 20 % of all KS statistics computed for orthologous groups (KS ≥0.1). This reduced the set of genes used to train the random forest from 1161 to 103.

The R package ‘randomForest’ was used to build a forest of decision trees in an approach based on that of Wheeler *et al.* [47]. A random forest was built using 10  000 trees, sampling five genes for each node (mtry=5). The same model building process was repeated using a range of values of mtry and model accuracy (as measured by F1 score), did not change. Each tree was reconstructed using a bootstrap sampling of the strains, taking seven extraintestinal and seven gastrointestinal strains for each tree. The gene whose bitscore values best separated the two classes was selected as the first node in the decision tree, and assigned an optimal scoring cutoff for partitioning samples into daughter nodes. For each of the two groups resulting from the split, if another gene could further separate the two classes, this was included in the tree as well. For each node in the tree, a new sample of five genes was taken. Trees are reconstructed until no more information from the sampling of genes can improve discrimination between the two classes. Key indicator genes were selected based on their feature importance scores, which were calculated based on the decrease in accuracy of predictions caused by permuting the variable of interest in the test data [43]. To assess the significance of the variable importance measures, the random forest model was re-trained 100 times with the classes permuted each time, in an approach similar to that taken by Huynh-Thu *et al.* [49]. Permutations that resulted in the same phenotype allocation as the true dataset were re-run. Empirical *P*-values were computed by identifying the percentage of feature importance values generated using the permuted data that were higher than each true feature importance value. Genes of interest were identified by running the model building process with the true classes 100 times and identifying the genes that had a variable importance with an empirical *P*-value <0.1 in 75 or more models.

### Electron microscopy

Electron microscopy was performed by the Manawatu Microscopy and Imaging Centre (Massey University, Palmerston North, New Zealand). Bacteria were pelleted and fixed in Modified Karnovsky’s fixative in 0.1M phosphate buffer (pH 7.2) at 4 °C overnight. Following buffer washing, samples were passed through a syringe filter holder containing a nylon membrane to collect the cells (0.4 um pore size; Isopore, Merck Millipore). Bacteria-exposed filters were dehydrated with a graded ethanol series before critical point drying. Samples were mounted and gold sputter coated according to standard procedures. Images were captured on a Quanta 200 scanning electron microscope (FEI) using an accelerating voltage of 20 kV.

## Results

In this investigation we examined whether there were any significant genomic differences between *C. jejuni* isolated from extraintestinal and gastrointestinal environments. We found no significant difference in the overall gene content of the isolates tested, however, we identified differences in the burden of rare mutations in key genes that have been implicated in *Campylobacter* virulence. We found that the patterns of sequence variation we identified in our study are unlikely to have strong predictive value in identifying other strains of invasive *C. jejuni*, however, they are indicative of a shift in selection pressures that occurs during extraintestinal infection.

### No significant difference in gene content between extraintestinal and gastrointestinal isolates

**Fig. 1.**
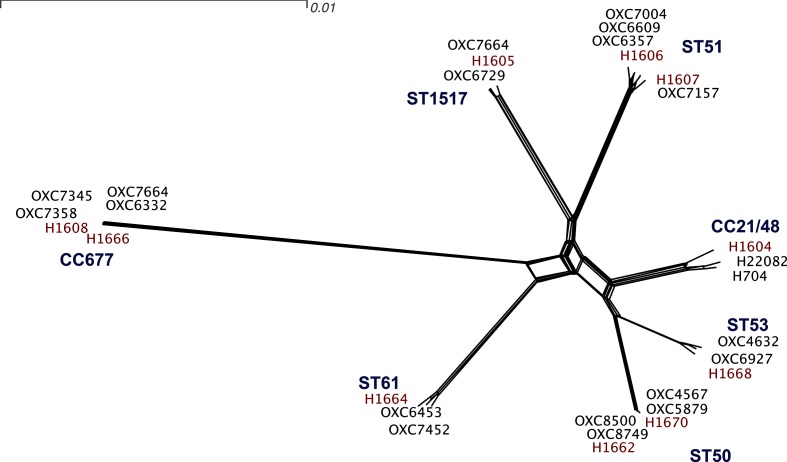
NeighborNet diagram of the genetic similarity of strains included in the study. The scale bar indicates 0.01 substitutions per site in a core gene alignment. Invasive strains are highlighted in red type. Each invasive strain was paired with two gastrointestinal strains, shown in black type, from the Oxfordshire surveillance project from the same clonal complex. Clonal complexes are highlighted in bold blue type.

A NeighborNet diagram of relatedness of the strains used in our study indicated that the randomly selected ST50 strains we included in our analysis were distantly related to the New Zealand invasive isolates, and further analysis of the ST50 clade indicated that our strains were members of a rare subgroup (Fig. S1). After inclusion of gastrointestinal representatives from this smaller clade we were able to confirm that each invasive strain was closely related to the selected reference strains, and invasive strains from the same clonal complex were also closely related to each other, but that the clonal complexes (CCs) and STs included in our study were genetically distinct from each other (Fig. 1). The overall genome size of the extraintestinal isolates was similar to that of the gastrointestinal isolates examined (1.655 Mb±13 kb vs 1.659 Mb±28 kb), as was the number of predicted genes (1729±22 vs 1745±42). GC content was also indistinguishable (30.34±0.05 % vs 30.36±0.06 %). We found no clear evidence of gene presence/absence patterns associated with invasive infection (Table S2).

### A subset of genes show an increased burden of rare mutations in invasive strains

Given that there were no differences in overall genome content between gastrointestinal and extraintestinal strains, we scanned the genomes for differences in mutational burden across genes using the DeltaBS approach [24] and a random forest classifier [47]. The DeltaBS approach uses profile hidden Markov models [50] to take a diverse collection of sequences of the same gene from different organisms and capture patterns of sequence variation that occur commonly in nature (in this case, within the *Epsilonproteobacteria*). These models are then used to score the protein coding genes of these strains to produce bitscores, which indicate how well each protein conforms to modelled sequence constraints on that gene. The distribution of these bitscores can then be used by the random forest to identify genes which show a difference in adherence to sequence constraints between strains from the two infection niches. A difference in bitscore distribution is likely to be caused by a change in selective pressures on a gene in one niche, leading to increased accumulation of mutations not commonly observed in nature (called rare or uncommon mutations in this manuscript, for brevity). This accumulation of rare mutations is likely to result in the degradation of the gene, however, these mutations could also result in a change to the function or structural stability of the protein. For a given protein family, the difference in bitscore for one strain and the median bitscore for the strain collection (DeltaBS) can be used as an indication of the likelihood that this accumulation of mutations is deleterious [24].

**Fig. 2.**
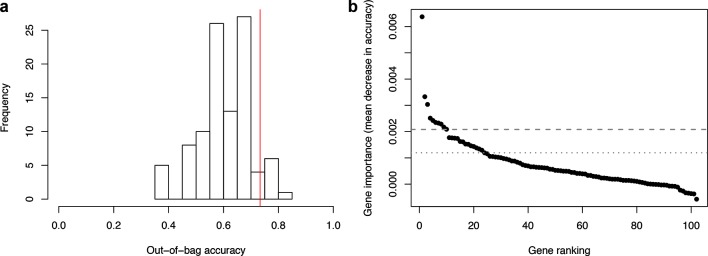
A random forest model identifies a subset of genes that show increased sequence divergence in invasive isolates (a): Performance of the random forest model compared with that of 100 models built using permuted labels. Accuracy of the classifier built using true classes is indicated with a red line, and accuracies for the permuted models are shown as a histogram. (b): Feature importance for each gene in a representative random forest model. Grey lines correspond to empirical *P*-values of 0.05 (dashed line) and 0.1 (dotted line), calculated using feature importance values derived from permuted data.

To assess the value of the genes we identified, we compared the performance of the random forest classifier trained to discriminate extraintestinal and gastrointestinal strains with those of classifiers built using permuted classes (randomized assignments of extraintestinal and gastrointestinal labels in the same ratios as the true classes). Out-of-bag (OOB) estimates of error can be used to assess the accuracy of a random forest predictor by testing the prediction accuracy of each tree in the forest on previously unseen data. The OOB estimate of error for the true classes was 26.7 . Of the models built using permuted labels 18 % had error rates this low (Fig. 2a). This 26.7 % overall error rate included 70 % error in identifying extraintestinal strains (Table S3). These results indicate that the patterns associated with invasiveness identified in our study do not generalize well across the different isolates, owing to weak convergence in the patterns of mutational burden observed. While the model did not have high predictive accuracy, examining the most informative genes identified by the model can give us insight into the genomic features that best separate the two classes. A set of 21 genes were identified that met the selection criterion (Fig. 2b and Table 1). Sequences were manually inspected, and one gene (*pseA*) was excluded as a false positive due to incorrect ortholog calling by Roary, and two others (*purE* and *group_904*) were excluded for only showing unusual patterns of sequence variation in ST51. Re-building the model with a training dataset that excluded these genes did not change the variable importances of the remaining candidates.

**Table 1.** Genes with the greatest value in discriminating between invasive and gastrointestinal *Campylobacter* Reference locus ID refers to the locus ID for the homologous sequence in *Campylobacter jejuni* ATCC 700819. VI: variable importance, as measured by mean decrease in classification accuracy upon permuting a variable. % top: percentage of models that listed this gene among its top predictors.

**Gene**	**Ref. locus ID**	**Description**	**Mean VI**	**VI *P*-value**	**% top**
*mreB*	Cj0276	Rod shape-determining protein MreB	0.007	0.011	100
*msbA*	Cj0803	Lipid export ABC transport protein	0.003	0.026	100
*nuoL*	Cj1568c	NADH dehydrogenase subunit L	0.003	0.027	100
*htpX*	Cj0723c	Peptidase, M48 family protein	0.003	0.036	100
*group_728*	Cj1236	COG1565: Uncharacterized conserved protein	0.002	0.038	100
*nrfA*	Cj0584	Cytochrome c552 precursor	0.002	0.040	100
*group_922*	Cj1357c	DNA polymerase III subunit delta'	0.002	0.040	100
*gltD*	Cj1345c	Glutamate synthase [NADPH] small chain	0.002	0.046	100
*pgp1*	Cj1706c	Purine nucleoside phosphorylase	0.002	0.048	100
*rplD*	Cj0612c	LSU ribosomal protein L4p (L1e)	0.002	0.050	100
*cft*	Cj1476c	Non-heme iron-containing ferritin	0.002	0.054	100
*porA_3*	Cj1572c	Pyruvate-flavodoxin oxidoreductase	0.002	0.057	100
*nuoH*	Cj1131c	NADH-ubiquinone oxidoreductase chain H	0.002	0.062	99
*group_3265*	Cj0041	Xaa-Pro aminopeptidase	0.002	0.066	97
*galE*	Cj0653c	UDP-glucose 4-epimerase	0.002	0.067	99
*fliK*	Cj0002	Flagellar hook-length control protein FliK	0.002	0.067	95
*rplL*	Cj0477	LSU ribosomal protein L7/L12 (P1/P2)	0.002	0.069	96
*gltX*	Cj1288c	Glutamyl-tRNA synthetase	0.001	0.069	94
*dnaN*	Cj0002	DNA polymerase III subunit beta	0.001	0.073	94
*selU*	Cj0500	tRNA 2-selenouridine synthase	0.001	0.082	86
*group_537*	Cj0530	Periplasmic protein	0.001	0.081	84

There were five major, interrelated themes that emerged in the functions of the genes identified. We identified a number of genes which have previously been linked with virulence, LPS biosynthesis, cell shape, stress response and iron acquisition. *htpX* is not well documented in *Campylobacter jejuni*, but is a known virulence gene whose inactivation affects adhesion, cell morphology and surface antigens in *Streptococcus gordonii* [51]. The gene is upregulated in *C. jejuni* in a piglet stomach model [52]. Three unique mutations were observed in H1664 and several mutations were shared by H1606, H1607 and one of the Oxfordshire strains from the same sequence type.

Both *msbA* and *galE* are involved in lipopolysaccharide biosynthesis. *msbA* carried the same mutation in both invasive members of ST50 and *galE* carried the same mutation in both invasive members of ST51, indicating that these are likely to be lineage effects, and therefore don’t show evidence of convergent evolution. *msbA* functions as a flippase of the lipid-A core oligosaccharide, transporting it from the inner to the outer cell membrane [53]. Mutation of the gene has led to an elongated rod morphology in *Eschericiha coli* [54], and a mutant in *Neisseria gonorrhoeae* showed a growth defect with less LPS and more phospholipids in the membrane [55]. The *galE* gene provides Gal and GalNAc for the three major cell-surface carbohydrates: lipopolysaccharides, capsule and glycoprotein N-linked heptasaccharide [56]. *galE* also has a role in virulence [57], and a *C. jejuni* mutant of the gene has been found to be impaired in its ability to adhere to and invade intestinal cells, but still colonized at wild-type levels [57]. The enzyme is thought to be required for synthesis of the polysaccharide capsule of *C. jejuni*, which modulates the host immune response [56, 58].

**Fig. 3.**
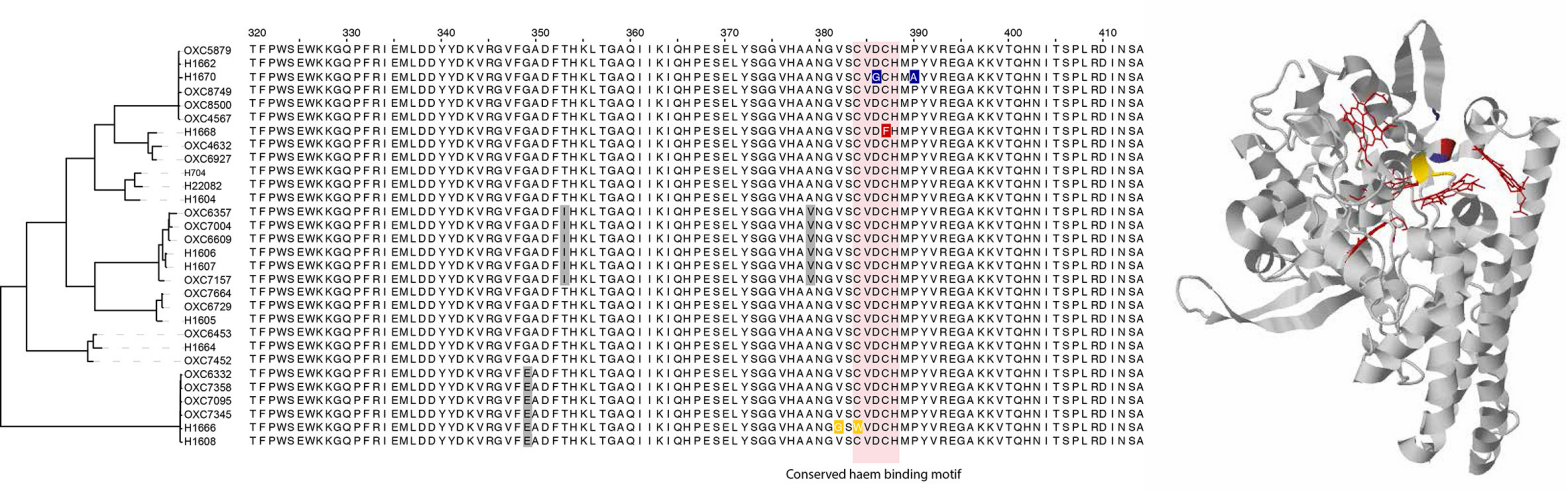
Partial sequence alignment highlighting unique mutations in *nrfA* in invasive strains, compared with lineage-specific mutations observed in other regions. The sequence alignment was visualised using Jalview (http://www.jalview.org) and coloured by BLOSUM62 score. The maximum-likelihood tree was reconstructed using RAxML (v.8.2.8) using the GTR-GAMMA model and 100 bootstraps. The PDB structure 1FS7 was visualised using Jmol (www.jmol.org). The highlighted residues are the only unique mutations in sequence alignment and occur in a conserved haem binding site. Haem molecules are highlighted in red.

Members of the genus *Campylobacter* prefer a microaerobic environment [59], and are relatively intolerant of environmental perturbations compared with other pathogens [60]. Among the top genes affected by rare mutations were stress-response genes that coded for iron-containing proteins. *nrfA* is a cytochrome c552 precursor, with a role in nitrosative stress survival [61]. Human patients with *C. jejuni*-induced diarrhea produce elevated levels of nitric oxide, so the ability to tolerate nitrosative stress is thought to be a key feature of pathogenesis for this microbe [62]. The gene is up-regulated in *C. jejuni* in the pig stomach [52], and a *nrfA* mutant of *C. jejuni* has been shown to be defective for colonization in a chick model [63]. We observed unique mutations in isolates H1666, H1668 and H1670, all clustered in the same small area of the gene, a conserved haem binding region [64] (Fig. 3). Three out of ten invasive isolates carrying mutations not seen in any other strain in the study vs none out of 20 gastrointestinal isolates is significant according to a Fisher's exact test (*P*=0.03). However, the significance of these results does not come purely from the number of mutations, but also from their predicted functional effects – if most mutations don't have a dramatic impact on function, the bitscore distribution for each gene sequence is tightly clustered around the median value. Outliers from this bitscore distribution indicate a mutation that the profile HMM for that gene family has penalised heavily, because an amino acid with those chemical and structural features is rarely observed in that position in nature, usually because it is selected against. Two of these strains have incurred two nonsynonymous mutations in the same site, for a total of five mutations within nine amino acids in a conserved region of the protein, which is highly unusual. The DeltaBS values for these three mutations are 6.2, 9.3 and 11.3, which are extremely high values for proteins that haven't accumulated any insertions or deletions (see [24], Fig. 1C, for an indication of typical DeltaBS values for inactivated proteins). The DeltaBS values for the rest of the strains range between −0.3 and 2.5, which are consistent with what we expect for functional protein sequence variants. *gltD* is a glutamate synthase gene which contains an iron–sulfur complex. Knockout of this gene causes reduced growth under high osmolar conditions [65]. *gltD* carried unique mutations in isolates H1606, H1608 and H1666, as well as some gastrointestinal strains, however the unique mutations in the invasive strains were predicted to have a greater functional effect. Another protein containing an iron–sulfur complex in our analysis was *porA_3*, a pyruvate–flavodoxin reductase. This gene is involved in oxidative stress tolerance and redox [66]. The gene had unique mutations in isolates H1604, H1605, H1608 and H1664. *cft* is a non-heme iron-containing ferritin. The gene provides protection against oxidative stress, and its mutation increases susceptibility to killing by peroxide [67]. The gene is generally well-conserved across our strains, except for unique mutations in isolates H1608, H1664 and H1670. *mreB* and *pgp1* mutations have previously been linked with changes in cell morphology. MreB forms helical tracts down the length of the cell and positions peptidoglycan synthesis machinery [68]. Reduced expression of *mreB* results in a spherical phenotype in *C. jejuni* [69], and alteration of the protein results in morphological variation in other species [70–72]. The protein has a role in adherence to host cells in *E. coli* [73], and is upregulated in *C. jejuni* in response to mucins secreted by the human gut [74]. Variation in the gene has also been associated with both adaptation to new conditions [75, 76], and localization of virulence proteins in the cell [77–79]. The *mreB* gene has acquired unique mutations in four invasive strains, H1662, H1666, H1668 and H1670. Phase variation in *pgp1* has previously been shown to generate a rod-shaped morphology in *C. jejuni* [80, 81], and knockout of the gene results in a loss of pathogenicity and loss of ability to effectively burrow through intestinal mucus in a mouse model [82]. Phase variation in *pgp1* was observed in one gastrointestinal ST50 isolate, but *pgp1* also carried unique nonsynonymous substitutions in H1604 and H1664 and a truncation in H1664. Similar to *pgp1*, *pgp2* is also involved in mediating cell morphology and deletion has been shown to cause a switch to rod-shaped morphology and defects in motility and biofilm formation, as well as impairment in a chick colonization model [83]. Because we identified mutations in both *mreB* and *pgp1*, we examined the sequences for this gene as well. One invasive strain carried a nonsynonymous mutation deemed by deltaBS to have a potentially functional effect. This strain did not carry unusual sequence variants in *mreB* or *pgp1*. Overall, eight out of ten invasive isolates showed rare mutations in either *mreB, pgp1* or *pgp2*, compared with four out of 20 gastrointestinal isolates (see Figs, S2–S4 for sequence alignments).

In addition to fitting themes consistent with plausible adaptive mechanisms for invasiveness in *Campylobacter*, some of our genes have been identified as relevant to pathogenesis by other studies. Garénaux *et al*. [66] assayed the response of NCTC 11168 to oxidative stress caused by paraquat, and found that *porA_3*, *cft* and *mreB* were overexpressed under these conditions. Hyytiäinen *et al*. [84] also showed that *porA_3* and *nuoM* are both downregulated in response to ciprofloxacin. In a study of the abortifacient sheep clone IA 3902 [85] *flgK* and *nuo* genes were expressed at a higher level than in NCTC 11168 and iron acquisition genes were expressed at a lower level. Further study also identified upregulation of *nrfA* in this strain [86]. A study of the genes involved in response to acidic pH and stomach transit [52] identified changes in the expression of *htpX*, *nrfA* and *cft* as important for stomach transit and *mreC*, which is also involved in cell shape, as influenced by acid shock.

### Changes in cell shape in MreB and Pgp1 mutants

**Fig. 4.**
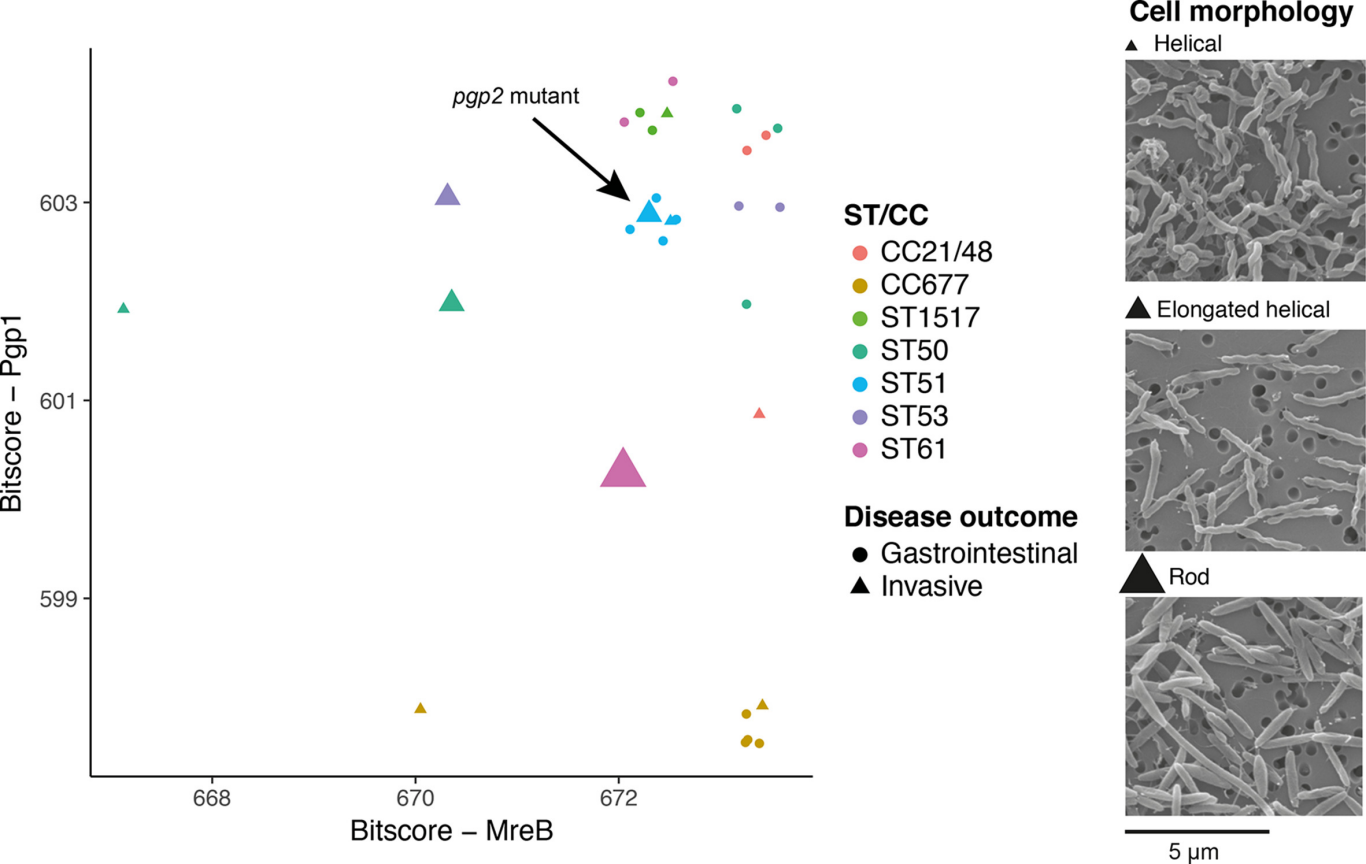
Sequence changes in cell shape genes relate to changes in cell morphology. Bitscore values are plotted for extraintestinal and gastrointestinal isolates for two cell-shape-determining proteins identified as associated with invasiveness which appeared in the top scoring genes. High bitscores correspond to strong agreement between individual protein sequences and modelled sequence constraints for that gene. Large differences in bitscore within an ST indicate the accumulation of a mutation/mutations that are not frequently observed in that gene within the *Epsilonproteobacteria*. The position of the points is offset slightly (up to 0.3 bits) to allow discrimination of strains with identical scores for each gene. Electron micrographs for representative invasive isolates from this study are shown to illustrate the cell morphologies described. Size scale is the same for each image. Note: one ST50 sample has been excluded from the plot because its score for *pgp1* was much lower and distorted the figure.

To test whether the unusual sequence variants of *mreB*, *pgp1* and *pgp2* resulted in morphological changes, we performed scanning electron microscopy on all invasive isolates, as well as other gastrointestinal isolates from the same sequence type, collected at a similar time in New Zealand (see Methods). We found morphological changes in four out of ten invasive isolates in our collection. Strain H1664 had a rod-shaped morphology, which is likely to be attributable to a truncation of *pgp1*, while strains H1607, H1662 and H1668 had an elongated helical phenotype. These cell shape changes were not observed in the extraintestinal isolates in our study that carried typical sequence variants of *mreB*, *pgp1* and *pgp2* (Fig. 4), or in the gastrointestinal isolates.

## Discussion

*Campylobacter* is the most common cause of enteric infection in the world, but only rarely causes extraintestinal infection. It is still unclear whether there are any specific genetic factors that make strains more likely to cause systemic infection. Genetic drivers of invasiveness have been identified in *Neisseria meningitidis* [87], *Streptococcus pneumoniae* [88] and Group A streptococci [89]. Other comparative analyses have successfully identified genetic differences leading to differences in survival through the food processing chain [90] and differences in host tropism [91] in *Campylobacter*. This study aimed to address whether there was a common genetic signature associated with adaptation to an invasive lifestyle in a sampling of *Campylobacter jejuni* isolated from extraintestinal infections in New Zealand.

If there is a single evolutionary path to extraintestinal infection in *Campylobacter* involving strong selection on key genes, we would expect this to be reflected in convergence on the same gene loss/gain or mutational events, however, if multiple independent routes to achieving extraintestinal infection exist, or if selective pressures on specific genes are weak, this signal is likely to be difficult to identify in such a small sampling of isolates. Other confounding factors, such as host susceptibility and microbiota, are also likely to play a role in infection outcomes, further complicating the link between genotype and infection outcome. The random forest built in our study achieved better accuracy in predicting infection outcome with real rather than permuted labels, but failed to identify consistent signatures of sequence variation present in all extraintestinal isolates in the study, indicating that the genomic signatures identified in our study do not occur consistently enough to be used to confidently identify other invasive isolates of *C. jejuni*.

Some of the genes that have been identified as top predictors of invasive infection have been linked with infection mechanisms in the existing literature. We discovered that mutations in two genes that control cell shape and adhesion to host cells when considered collectively were effective at separating six invasive strains from the two major sequence spaces occupied by the remaining strain collection, and that three of these six showed an observable loss of their tight helical morphology. These morphological changes could be adaptation to a new infection mechanism, or a response to increased stress caused by the shift of a gastrointestinal pathogen to a new environment. Cell shape has been tied to pathogenicity in the past, with the helical shape of cells of members of the genus *Campylobacter* specifically implicated in colonization ability [79, 80, 92, 93]. The helical shape of members of the genus *Campylobacter* is thought to aid in burrowing through the mucus layer of the gut [82, 94], however, rod-shaped mutants are frequently observed during passage in the laboratory [95] or in a chicken host [96], partly owing to phase variation causing phenotypic plasticity within a strain, which is thought to improve the long-term survival potential of the population [80]. While cell shape switching has been observed in the past due to phase variation in *pgp1* and *pgp2* [81], the mutations identified in our study are amino acid substitutions in the case of the elongated helical variants and a premature stop codon in the case of the rod-shaped variant, indicating that they would be less prone to reversal. An *in vivo* selection experiment performed by Field *et al*. [97] yielded rod-shaped strains with increased ability to survive in an *in vivo* chick embryo model, indicating that this phenotype may emerge and be adaptive in the bloodstream. Stahl *et al*. [82] showed that later stages of infection were not impaired in a rod-shaped *pgp1* knockout, but initial infection could not be achieved, indicating that this phenotype isn’t likely to be selected for by serial invasive infections. In this study, variants that had a curved-rod morphology could still burrow through mucus and achieve infection [82], indicating that our elongated helical variants could still be effective colonizers. *pgp1* mutants show no defect in adhesion to and invasion of human epithelial and macrophage cells *in vitro*, and in fact survive slightly longer than wild type strains [80, 93]. In addition, changes in cell shape and an alteration in peptidoglycan structure resulting from *pgp1* and *pgp2* mutations also altered levels of activation of human host cell receptors in response to infection [80, 93], indicating a possible advantage of the change in cell shape. An isolate showing a similar elongated helical morphology to that observed in our study, arising from mutation in a different gene (*Cj1564*), showed decreased ability to migrate through agar and decreased ability to adhere to and invade Caco-2 cells, however, it showed no impairment in chick colonization [83]. These findings collectively indicate that cell shape variation could be adaptive in the systemic niche, though ability to colonize a new host could potentially be compromised.

While some of the key indicator genes identified in this study are promising, we are cautious about interpreting the results of our study, as we have a small sampling of invasive isolates from which to identify trends. While the random forest model performed well in permutation testing, the likelihood that the model would be predictive in a separate set of invasive isolates is low, due to the small number of informative genes identified and the limited classification accuracy for the invasive class. A limitation of our study is that we have compared invasive isolates of *Campylobacter* from New Zealand with gastrointestinal isolates from the United Kingdom, which could introduce some confounding in patterns of sequence variation due to country of isolation. The Oxford Surveillance project provided greater coverage of isolates that were closely related to our invasive cases, while isolates and whole-genome sequences for representatives from human cases of gastroenteritis in New Zealand were only available for a small number of matching sequence types. The isolates from Oxford were therefore chosen to maximize our power to detect any associations.

### Concluding statements

Our findings add to a growing body of literature linking cell shape and virulence in members of the genus *Campylobacter* [80, 82, 83, 93]. To the best of our knowledge, this is the first reported finding of this cell shape change occurring in a human host, however similar changes have been noted in members of the genus *Campylobacter* under stress conditions [98] and during passage through a chicken host [96]. The helical cell shape of the members of the genus *Campylobacter* is thought to be important for the passage of cells through the mucus layer of the gut [82], so these findings raise the question of whether this ability is not required for systemic infection, or whether this cell shape change is a response to the stress of a new environment and would prevent transmission to a new host. Given support in the literature for an association between invasiveness and lipopolysaccharide biosynthesis, stress response and cell shape, the candidates identified in this study are worth investigating further for their role in invasive infection. The workflow used to perform this analysis is straightforward and requires little manual intervention to perform, therefore similar investigations could be repeated with other collections of invasive pathogens, allowing for cross-validation of results. The code for performing this analysis is freely available at https://github.com/Gardner-BinfLab/invasive_campylobacter.

## Data bibliography

1. Blackmore T., Reynolds A. D., Midwinter, A. C., Marshall, J., French, N. P., Biggs, P. J. Sequence Read Archive BioProject PRJNA475221 (2018).
2. Wheeler, N. E., Marshall, J. GitHub https://github.com/Gardner-BinfLab/invasive_campylobacter (2018).
