# Supplementary material for: Genomic correlates of extraintestinal infection are linked with changes in cell morphology in Campylobacter jejuni
Source: Microb Genom. 2019 Feb 19;5(2):e000251. doi: 10.1099/mgen.0.000251 (PMC6421344; doi:10.1099/mgen.0.000251)
Supplement: Supplementary data [file mgen-5-251-s001.pdf]

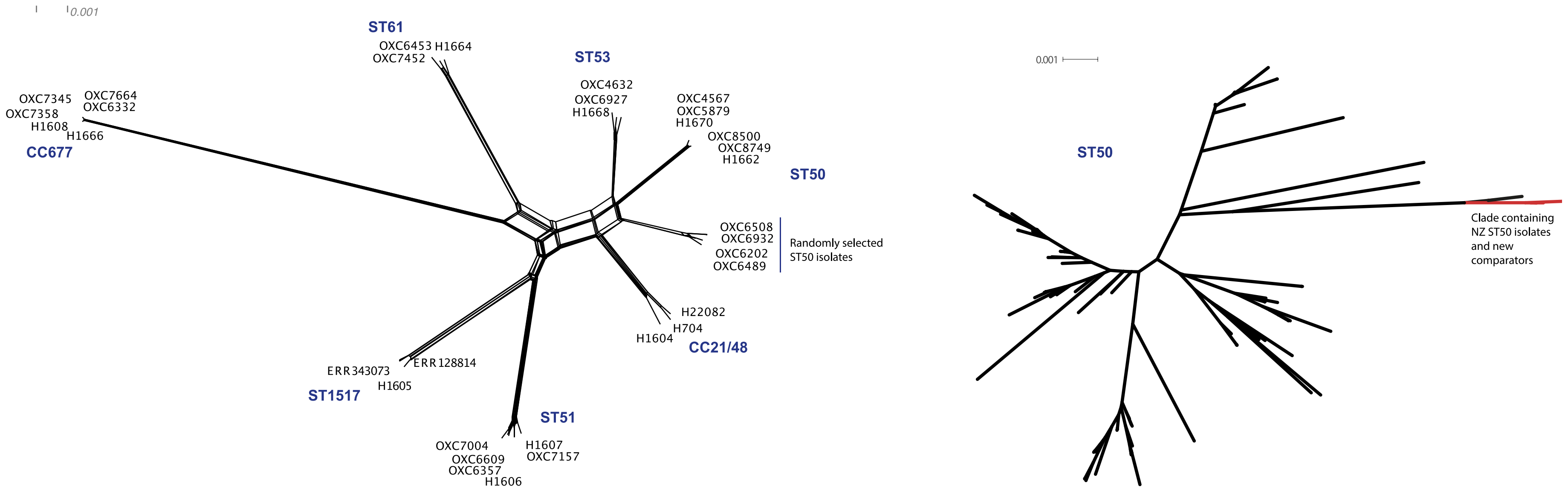

Supplementary Figure 1 | Phylogenetic relationships between all strains considered for this study

A NeighborNet diagram illustrates the distant relationship between the randomly selected ST50 strains and the New Zealand ST50 strains, while the tree on the right illustrates the ST50 phylogeny from which closer comparator strains were selected. NeighborNet diagram was generated by SplitsTree and the maximum likelihood tree was generated using RAxML (v.8.2.8) using the GTR-GAMMA model and 100 bootstraps.

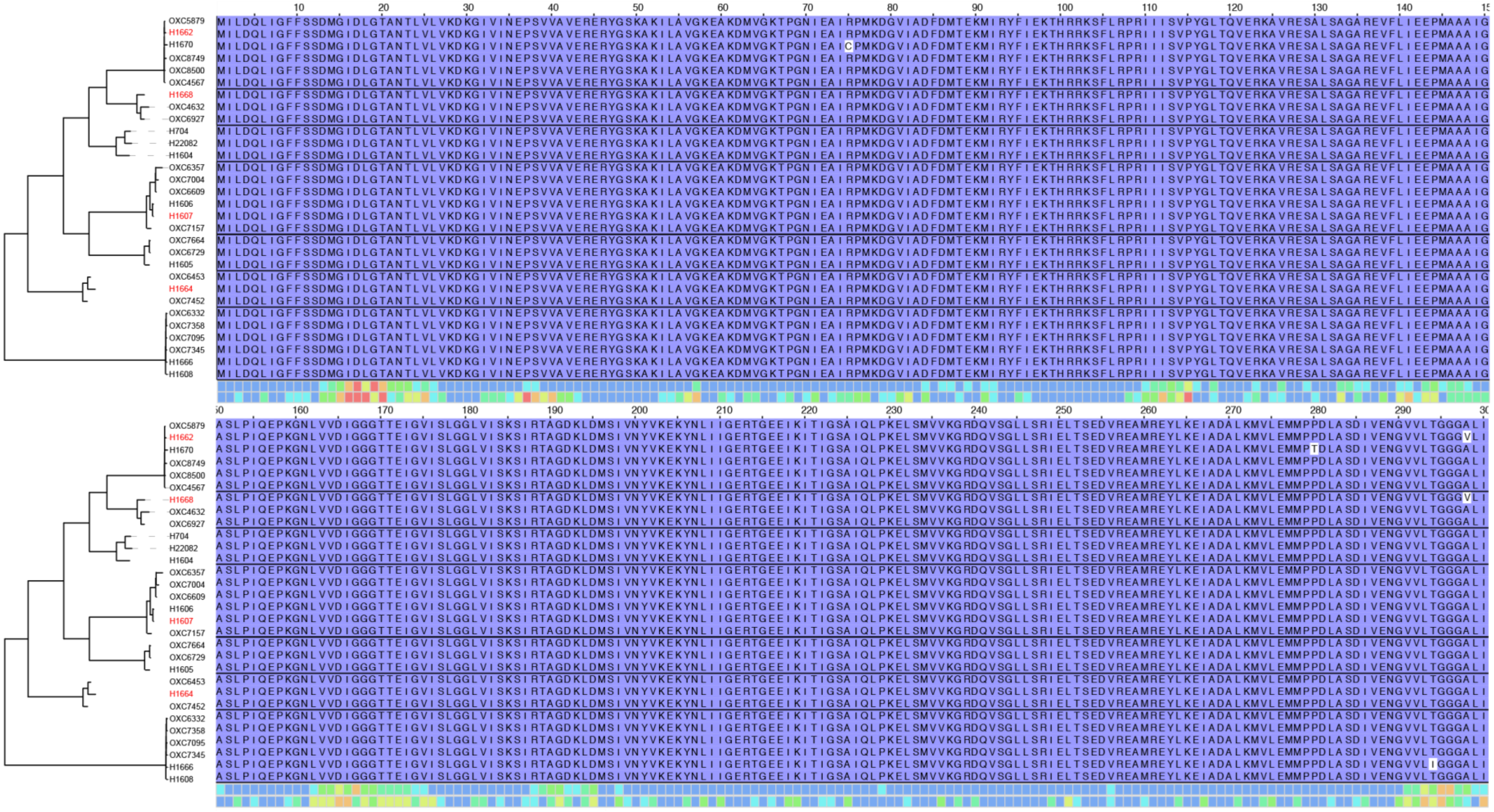
OXC5879
H1662
H1670
OXC8749
OXC8500
OXC4567
H1668
OXC4632
OXC6927
H704
H22082
H1604
OXC6357
OXC7004
OXC6609
H1606
H1607
OXC7157
OXC7664
OXC6729
H1605
OXC6453
H1664
OXC7452
OXC6332
OXC7358
OXC7095
OXC7345
H1666
H1608

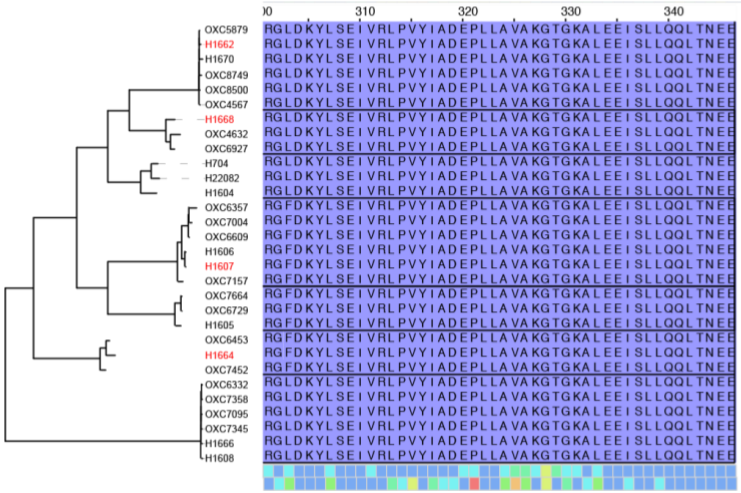

Supplementary Figure 2 | Sequence alignment of mreB

Sequence alignment was visualised using Jalview (http://www.jalview.org) and coloured by BLOSUM62 score. The maximum likelihood tree was generated using RAxML (v.8.2.8) using the GTR-GAMMA model and 100 bootstraps. Strains that show morphological changes are highlighted in red. Panels running below the sequence indicate sequence conservation (top) and predicted mutational robustness (bottom) calculated using the Phyre2 web server (Kelley, L. A., Mezulis, S., Yates, C. M., Wass, M. N., & Sternberg, M. J. E. (2015). The Phyre2 web portal for protein modeling, prediction and analysis. *Nature Protocols*, *10*(6), 845–858.)

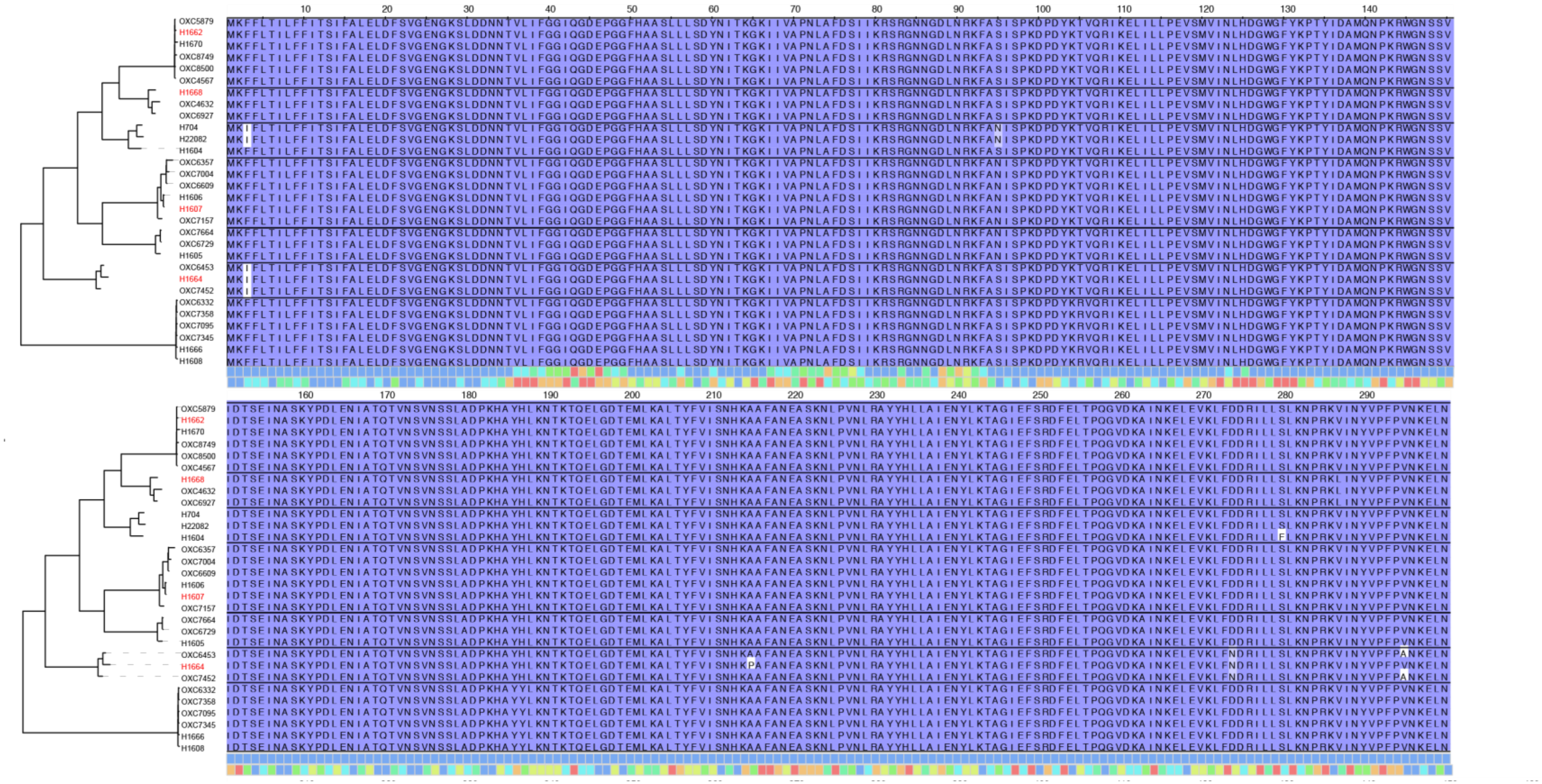
OXC5879
H1662
H1670
OXC8749
OXC8500
OXC4567
H1668
OXC4632
OXC6927
H704
H22082
H1604
OXC6357
OXC7004
OXC6609
H1606
H1607
OXC7157
OXC7664
OXC6729
H1605
OXC6453
H1664
OXC7452
OXC6332
OXC7358
OXC7095
OXC7345
H1666
H1608

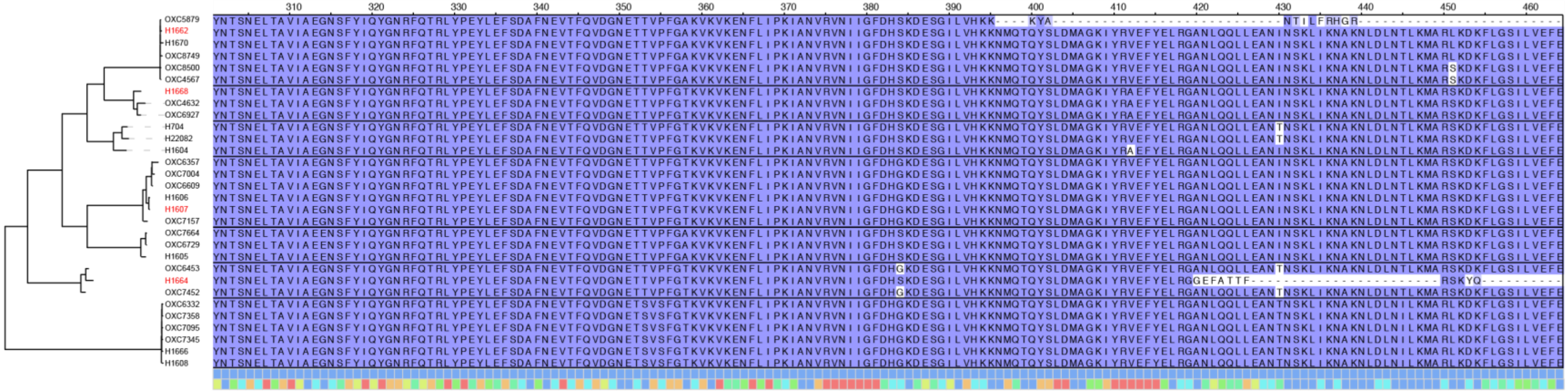

Supplementary Figure 3 | Sequence alignment of pgp1

Sequence alignment was visualised using Jalview (http://www.jalview.org) and coloured by BLOSUM62 score. The sequence for strain OXC5879 is truncated by a previously seen A insertion in an 8-A tract (Esson *et al.* 2016). The maximum likelihood tree was generated using RAxML (v.8.2.8) using the GTR-GAMMA model and 100 bootstraps. Strains that show morphological changes are highlighted in red. Panels running below the sequence indicate sequence conservation (top) and predicted mutational robustness (bottom) calculated using the Phyre2 web server (Kelley, L. A., Mezulis, S., Yates, C. M., Wass, M. N., & Sternberg, M. J. E. (2015). The Phyre2 web portal for protein modeling, prediction and analysis. *Nature Protocols*, *10*(6), 845–858.)

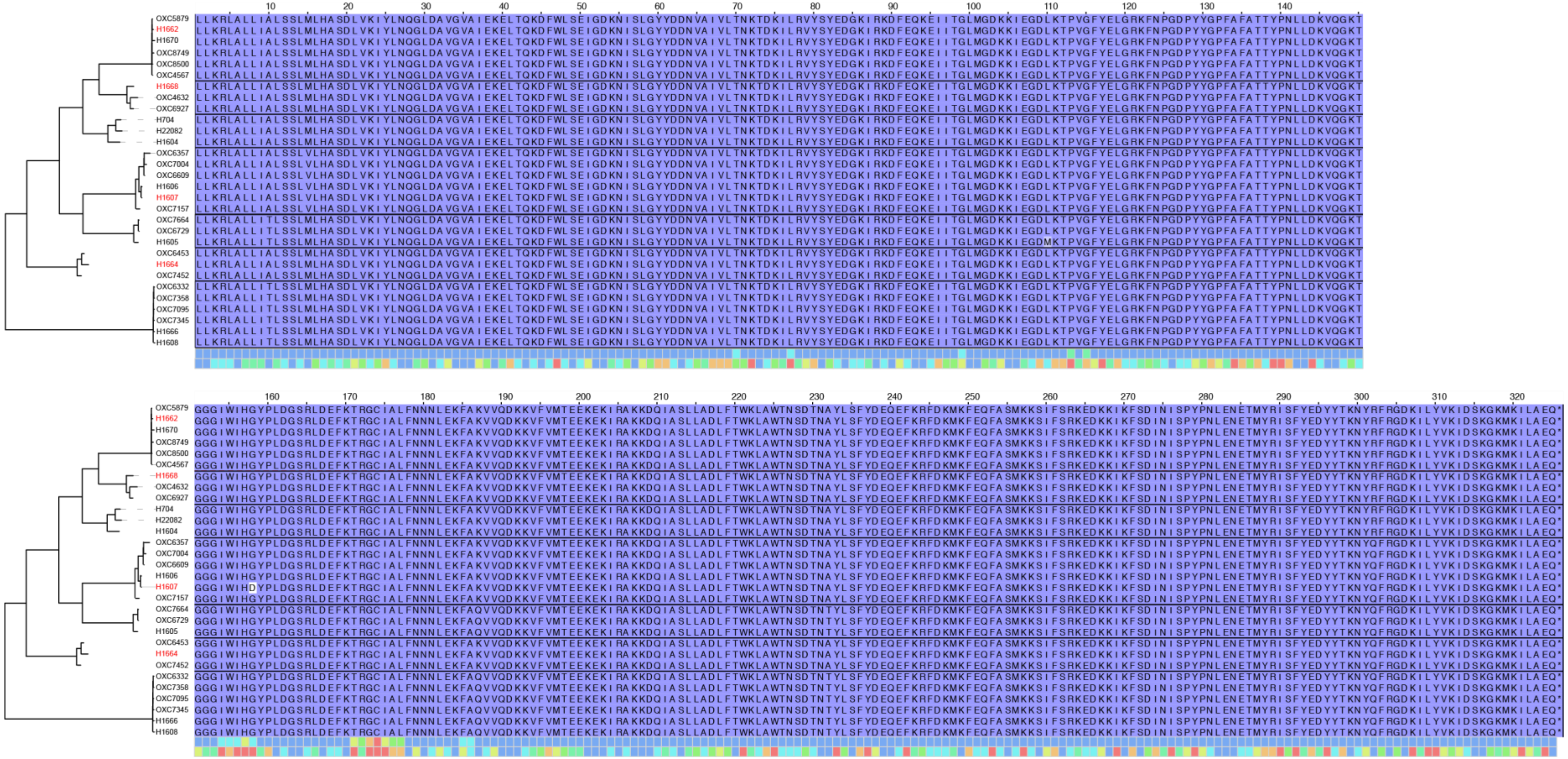

Supplementary Figure 4 | Sequence alignment of pgp2

Sequence alignment was visualised using Jalview (http://www.jalview.org) and coloured by BLOSUM62 score. The maximum likelihood tree was generated using RAxML (v.8.2.8) using the GTR-GAMMA model and 100 bootstraps. Strains that show morphological changes are highlighted in red. Panels running below the sequence indicate sequence conservation (top) and predicted mutational robustness (bottom) calculated using the Phyre2 web server (Kelley, L. A., Mezulis, S., Yates, C. M., Wass, M. N., & Sternberg, M. J. E. (2015). The Phyre2 web portal for protein modeling, prediction and analysis. *Nature Protocols*, *10*(6), 845–858.)

Supplementary Table 1 | Isolates included in this study

Strain name	Accession	ST	Disease	Year	Country
**OXC4567**	ERR278423	ST50	Gastroenteritis	2009	UK
**OXC8749**	ERR713656	ST50	Gastroenteritis	2014	UK
**OXC8500**	ERR494558	ST50	Gastroenteritis	2013	UK
**OXC5879**	ERR593768	ST50	Gastroenteritis	2011	UK
**OXC6357**	ERR083973	ST51	Gastroenteritis	2011	UK
**OXC6609**	ERR108372	ST51	Gastroenteritis	2011	UK
**OXC7157**	ERR193205	ST51	Gastroenteritis	2012	UK
**OXC7004**	ERR221288	ST51	Gastroenteritis	2012	UK
**OXC6927**	ERR356128	ST53	Gastroenteritis	2012	UK
**OXC4632**	ERR278461	ST53	Gastroenteritis	2009	UK
**OXC6453**	ERR084064	ST61	Gastroenteritis	2011	UK
**OXC7452**	ERR278359	ST61	Gastroenteritis	2012	UK
**H22082**	SRR065736	CC48	Gastroenteritis	2005	NZ
**H704**	SRR7516982	CC48	Gastroenteritis	2008	NZ
**OXC7095**	ERR221374	CC677	Gastroenteritis	2012	UK
**OXC7358**	ERR278242	CC677	Gastroenteritis	2012	UK
**OXC6332**	ERR083948	CC677	Gastroenteritis	2011	UK
**OXC7345**	ERR278230	CC677	Gastroenteritis	2012	UK
**OXC7664**	ERR343073	ST1517	Gastroenteritis	2012	UK
**OXC6729**	ERR128814	ST1517	Gastroenteritis	2011	UK
**H1604**	SRR7280458	CC21	Bacteremia	2011	NZ
**H1605**	SRR7280459	ST1517	Bacteremia	2010	NZ
**H1606**	SRR7280460	ST51	Bacteremia	2011	NZ
**H1607**	SRR7280461	ST51	Bacteremia	2010	NZ
**H1608**	SRR7280454	CC677	Bacteremia	2011	NZ
**H1662**	SRR7280455	ST50	Bacteremia	2011	NZ
**H1664**	SRR7280456	ST61	Bacteremia	2011	NZ
**H1666**	SRR7280457	CC677	Bacteremia	2010	NZ
**H1668**	SRR7280462	ST53	Bacteremia	2012	NZ
**H1670**	SRR7280463	ST50	Bacteremia	2010	NZ

Supplementary Table 3 | Confusion matrix built from predictions on training data made by the random forest model

		**Prediction**		**Classification error**
		Gastrointestinal	Invasive	
**Class**	Gastrointestinal	19	1	0.05
	Invasive	7	3	0.70
